# The Upsurge of Impact Factors in Pediatric Journals Post COVID-19 Outbreak: A Cross-Sectional Study

**DOI:** 10.3389/frma.2022.862537

**Published:** 2022-03-29

**Authors:** Pritish Mondal, Lauren Mazur, Lilly Su, Suparna Gope, Esther Dell

**Affiliations:** ^1^Department of Pediatrics, Penn State College of Medicine, Hershey, PA, United States; ^2^Department of Administration and Leadership, Indiana University of Pennsylvania, Indiana, PA, United States; ^3^Harrell Health Sciences Library, Penn State College of Medicine, Hershey, PA, United States

**Keywords:** impact factor, Eigenfactor, bibliometrics, SNIP, publication bias, COVID-19, open access journals

## Abstract

**Background:**

Impact factor (IF) is a quantitative tool designed to evaluate scientific journals' excellence. There was an unprecedented upsurge in biomedical journals' IF in 2020, perhaps contributed by the increased number of publications since the COVID-19 outbreak. We conducted a cross-sectional study (2018–2020) to analyze recent trends in standard bibliometrics (IF, Eigenfactor, SNIP) of pediatric journals. We also estimated reference and publication counts of biomedical journals since publication volume determines the number of citations offered and IF.

**Methods:**

Various bibliometrics of pediatric journals and reference/publication volumes of biomedical journals were compared between 2020 vs. 2019 and 2019 vs. 2018. We also compared open access (OA) and subscription journals' trends. Finally, we estimated IF changes in the journals of a different specialty, pulmonology.

**Results:**

The study included 164 pediatric and 4,918 biomedical journals (OA = 1,473, subscription = 3,445). Pediatric journals' IFs had increased significantly in 2020 [median (IQR) = 2.35 (1.34)] vs. 2019 [1.82 (1.22)] (Wilcoxon: *p*-value < 0.001). IFs were unchanged between 2018 and 2019. Eigenfactor remained stable between 2018 and 2020, while SNIP increased progressively. Reference/publication volumes of biomedical journals escalated between 2018 and 2020, and OA journals experienced faster growth than subscription journals. IFs of pulmonary journals also increased considerably in 2020 vs. 2019.

**Conclusions:**

We report an upsurge in pediatric journals' IF, perhaps contributed by a sudden increase in publication numbers in 2020. Therefore, considering this limitation, IF should be cautiously used as the benchmark of excellence. Unlike IF, Eigenfactor remained stable between 2018 and 2020. Similar changes in IF were also observed among the journals of another specialty, pulmonology.

## Introduction

JCR (journal citation report) impact factor (IF) is considered a standard quantitative tool designed to evaluate and compare the quality of scientific journals (Clarivate, 2021a). IF is estimated by the ratio of total citations received by a journal in a given year to the number of citable articles published in two previous years (Calculator Academy Team, 2021). Unlike IF, other bibliometrics such as the Eigenfactor score weighs citations depending on citing journal's ranking (EIGENFACTOR.org), while source normalized impact per paper (SNIP) score is adjusted to the variations among the different scientific fields (Elsevier, 2012). Moreover, Eigenfactor and SNIP incorporate data over the last five and three years, respectively.

The value and misuse of IF has been under scrutiny for several years (Bornmann et al., 2012). Readers often select an article based on the number of citations it has received and the prestige of the publishing journal (Lemke et al., 2021). Brown and Gutman compared the bibliometrics of occupational journals and concluded that metrics such as Eigenfactor, SNIP, and Scimago journal ranking should be used in addition to IF to evaluate journals' quality (Brown and Gutman, 2018). However, most of the research on bibliometrics was conducted prior to the COVID-19 outbreak (Kianifar et al., 2014; Yuen, 2018). The latest JCR was published in June 2021, based on the 2020 data, and defined an unprecedented upsurge in pediatric and other biomedical journals' IF. Several publishers have been avidly marketing this report (EDP Sciences, 2021; ESA, 2021; Weingarden, 2021). Since the number of citations determines the IF, a sharp increase in publication volume during the COVID-19 outbreak possibly led to an upsurge in the citations offered to the biomedical journals (Aviv-Reuven and Rosenfeld, 2021). Between 2012 and 2019, the number of open access (OA) journal publications had increased considerably from one to four million articles, influencing citation volumes (Morrison, 2021). However, comparative analysis between OA and subscription biomedical journals' recent trends has not been reported.

We conducted a cross-sectional study to estimate IF and other bibliometrics (IF ranking, Eigenfactor, and SNIP) of pediatric journals between 2018 and 2020 and compared the rate of changes in the bibliometrics between 2020 and 2019. The secondary objectives of our study were to compare the growth of biomedical journals in 2020 with the previous years and estimate the difference between OA and subscription journals' trends. Finally, we analyzed IF changes among the journals of a different specialty, pulmonology, to understand whether IF upsurge was specific to the field of pediatrics or a general trend.

## Materials and Methods

### Data Collection

We accessed the JCR (Clarivate, 2021a) through Penn State Library and collected data (the year 2018–2020) on IF, IF ranking, Eigenfactor scores (Web of Science Group, 2021), as well as SNIP of the pediatric journals (CWTS Journal Indicators, 2021) (Table 1). It was not feasible to estimate individual citation counts received by each of the 50,000+ articles published between 2018 and 2019 in 164 pediatric journals. However, an overall increase in the number of published references across the medical fields in 2020 would perhaps reflect an increased volume of citations offered to the pediatric journals. Hence, we collected data on total reference (citation) counts from all biomedical journals since citing articles could be from any specialty. We accessed the Scimago journal rank (SJR) database (SJR, 2021b) and assembled the reference and publication volume estimates (2018–2020), of all 'Web of Science' indexed biomedical journals (Clarivate, 2021b; SJR, 2021c).

**Table 1:** The characteristics of bibliometrics of pediatric journals and publication/reference counts in biomedical journals between the years 2018–2020.

	**2020**	**2019**	**2018**
	**Median (IQR)**	**Median (IQR)**	**Median (IQR)**
**Pediatric journals**			
Publication	113.5 (169)	111 (140)	103 (161)
Impact factor (IF)	2.35 (1.34)	1.82 (1.22)	1.89 (1.28)
IF (first quartile)	3.95 (2.09)	3.08 (1.29)	2.88 (1.29)
IF (second quartile)	2.57 (0.52)	2.08 (0.73)	2.04 (0.71)
IF (third quartile)	1.97 (0.29)	1.56 (0.35)	1.50 (0.70)
IF (fourth quartile)	1.17 (0.73)	0.93 (0.58)	0.88 (0.66)
Eigenfactor score (EF)	0.0040 (0.0056)	0.0040 (0.0055)	0.0041 (0.0059)
Source normalized impact (SNIP)	1.12 (0.59)	0.99 (0.50)	0.95 (0.52)
**Pediatric journals (rate of acceleration)**			
Δ-IF (%)	25.41 (24.97)	1.99 (23.38)	N/A
Δ-EF (%)	−1.03 (18.92)	−1.15 (17.95)	N/A
Δ-SNIP (%)	14.16 (20.38)	3.45 (22.52)	N/A
**Biomedical journals (BM)**			
Reference	3581 (5692)	3028 (4607)	2940 (4472)
Publication	105 (158)	90 (129)	88 (127)
Δ-Reference (%)	14.33 (51.10)	1.88 (32.96)	N/A
Δ-Publication (%)	11.69 (43.01)	1.65 (26.83)	N/A
**Pulmonary journals**			
Impact factor (IF)	3.29 (2.44)	2.72 (2.41)	2.73 (2.27)
Δ-IF (%)	25.60 (22.48)	1.59 (17.54)	N/A

*Delta (Δ) represents a variable's annual change in a given year, with respect to the previous year. For example, Δ-IF in 2020 was calculated with the following equation: [(IF2020–IF2019)/IF2019], and expressed as a percentage*.

*IQR, interquartile range; OA, open access; N/A, Delta (Δ) was not applicable for 2018, since we did not consider data from the year 2017 in the study analyses*.

### Data Analyses

a) Test of normality: Shapiro-Wilk test was used to test for the normal distribution of the data. Please review Supplementary Table 1 for additional details.
b) Pediatric journals: We compared the trends in bibliometrics between 2020 vs. 2019 and 2019 vs. 2018 (Table 2). We also estimated the rate of acceleration in IF, measured by annual changes in IF, with respect to the previous year's IF (expressed as Δ). For example, Δ-IF in 2020 was calculated with the following equation: [(IF2020–IF2019)/IF2019], and expressed as a percentage. We further compared Δ-IF 2020 and Δ-IF 2019 (Table 2). The pediatric journals were divided into four groups (from first to fourth quartiles), based on the 2020 IF score. For example, out of 164 pediatric journals, 41 journals with the highest IFs were included in the cohort of first quartile journals. We further compared the changes in IF (Δ-IF) between 2019 and 2020 among the four groups.
c) Biomedical journals: Reference and publication counts of biomedical journals were compared between 2020 vs. 2019 and 2019 vs. 2018. We also compared the rate of acceleration (Δ) between 2020 and 2019. Biomedical journals were further divided into primarily OA or subscription journals, following SJR website classification (SJR, 2021a). The growth (Δ) among the OA and subscription journals were compared between 2020 and 2019, respectively (Table 3).
d) Generalizability of the results: we collected data on pulmonary journals' IF from JCR 2021 to comprehend whether the recent changes in IFs are a general trend vs. applicable to the field of pediatrics only. Pediatric pulmonary journals were excluded to avoid an overlap between pediatric and pulmonary journals. IFs of pulmonary journals were compared between 2020 vs. 2019 and 2019 vs. 2018. The rate of acceleration in pulmonary journals' IFs (Δ-IF) in 2020 was also compared with 2019.
e) Choice of statistical tests: Since the data were not normally distributed, Wilcoxon signed-rank tests were used to compare between individual years' bibliometrics (paired difference between two measurements of a group) and Mann-Whitney test to compare between OA vs. subscription journals (difference in an outcome variable between two independent groups). We compared the changes in IF (Δ-IF) between four quartiles of pediatric journals with the Kruskal-Wallis test (difference in an outcome variable between three or more independent groups). We used IBM SPSS 27 for data analyses.

**Table 2:** The comparative analyses between two consecutive years' bibliometrics (2020 vs. 2019 and 2019 vs. 2018) using Wilcoxon signed-rank tests.

	**2020 vs. 2019**		**2019 vs. 2018**	
	**Z statistics**	***p*** **-value**	**Z statistics**	***p*** **-value**
**Pediatric journals**			
Publication counts/journals	−2.596	0.009	−1.446	0.148
Impact factor (IF)	−10.597	<0.001	−1.638	0.101
IF rank (percentile)	−1.941	0.052	−2.085	0.037
Eigenfactor score (EF)	−0.922	0.356	−1.746	0.081
Source normalized impact (SNIP)	−8.451	<0.001	−2.730	0.006
**Pediatric journals (annual changes)**			
Δ-IF (%)	−8.188	<0.001	N/A	N/A
Δ-EF (%)	−1.226	0.22	N/A	N/A
Δ-SNIP (%)	−4.076	<0.001	N/A	N/A
**Biomedical journals**			
Reference counts/journal	−27.936	<0.001	−6.053	<0.001
Publications counts/journal	−30.074	<0.001	−6.303	<0.001
Δ-References (%)	−14.971	<0.001	N/A	N/A
Δ-Publications (%)	−18.495	<0.001	N/A	N/A
**Pulmonary journals**			
Impact factor (IF)	−7.341	<0.001	−0.149	0.882
Δ-IF (%)	−6.240	<0.001	N/A	N/A

*P-values <0.05 is considered statistically significant. Delta (Δ) represents a variable's annual change in a given year, with respect to the previous year. For example, Δ-IF in 2020 was calculated with the following equation: [(IF2020–IF2019)/IF2019], and expressed as a percentage*.

*OA, open access, N/A, Delta (Δ) was not applicable for 2018, since we did not consider data from the year 2017 in the study analyses*.

**Table 3:** The characteristics of open-access vs. subscription biomedical journals between 2018 and 2020.

	**2020**		**2019**		**2018**	
	**OA**	**Subscription**	**OA**	**Subscription**	**OA**	**Subscription**
References/journal	2414 (3859.25)	4030.5 (6004.25)	2088.5 (3052.25)	3454 (4919)	1956 (2735.25)	3329.5 (4824.25)
Publications/journal	77 (117.25)	114 (168.25)	68 (97.25)	100 (139.25)	65 (87)	96 (136.25)
Δ-References	15.56 (56.20)	13.71 (48.74)	3.28 (44.26)	1.56 (29.39)	N/A	N/A
Δ-Publications	12.83 (47.00)	11.11 (41.33)	2.66 (36.19)	1.45 (23.60)	N/A	N/A
Reference counts ratio* (OA: Subscription)	0.51		0.45		0.39	
Published articles ratio* (OA: Subscription)	0.43		0.40		0.36	

Data are presented as median (IQR). The last two rows (*) represent total reference counts and published articles counts, respectively. Delta (Δ) represents a variable's annual change in a given year, with respect to the previous year. For example, Δ-IF in 2020 was calculated with the following equation: [(IF2020–IF2019)/IF2019], and expressed as a percentage.

*IQR, interquartile range; OA, open access; N/A, Delta (Δ) was not applicable for 2018 since we did not consider data from the year 2017 in the study analyses*.

*OA journals experienced a higher rate of growth compared to subscription journals*.

## Results

1. We collected data on 164 pediatric journals, 4,918 biomedical journals (OA = 1,473, subscription = 3,445), and 85 pulmonary journals, respectively. Shapiro-Wilk test demonstrated that the bibliometrics of pediatric journals and publication data of biomedical journals were not normally distributed (Supplementary Table 1).
2. Pediatric journals:

(a) The number of publications [median (IQR)] in 2018, 2019 and 2020 were 103 (161), 111 (140), and 113.5 (169), respectively. Publication counts were significantly different between 2019 and 2020; however, there was no difference between 2018 and 2019 (Tables 1, 2). The median (IQR) IF of Pediatric journals in 2020 was 2.35 (1.34), significantly higher compared to 2019 IF of 1.82 (1.22) (Wilcoxon test: *p*-value < 0.001). Other bibliometrics (IF ranking and Eigenfactor) except SNIP (Table 2) were unchanged between those two years. IF and Eigenfactor also remained unchanged between 2018 and 2019. IF of all four quartile journals (Table 1) increased considerably in 2020 compared to 2019 (Wilcoxon test: *p*-value < 0.001 for all four quartiles), indicating that the changes were not confined to the top-ranked journals only.
(b) The rate of changes in bibliometrics: Δ-IF and Δ-SNIP were significantly higher in 2020 compared to 2019 (Wilcoxon test: *p*-value < 0.001), reflecting a higher rate of acceleration in 2020; however, Δ-Eigenfactor remained stable (Table 2). The Kruskal-Wallis test demonstrated that Δ-IF were not statistically different among the four quartiles of pediatric journals in 2020 (Kruskal-Wallis H: 1.96, degree of freedom = 3, *p* = 0.58), or in 2019 (Kruskal-Wallis H: 1.27, degree of freedom = 3, *p* = 0.73).

3. Biomedical journals:

(a) Average reference and publication counts were progressively and significantly escalated between 2018 to 2020 (Tables 1, 2). Δ-references and Δ-publications in 2020 were significantly higher compared to 2019, reflecting a higher rate of acceleration in 2020 than in 2019.
(b) OA vs. subscription journals: OA journals (reference and publication count) experienced higher growth than subscription journals with every passing year (Table 3). In 2020, the rate of acceleration for reference counts (Δ-references) was also higher in OA than in subscription journals (Mann-Whitney: *p*-value = 0.013) however, Δ-publications were not statistically different (Mann-Whitney: *p*-value = 0.38) during the same period.

4. Pulmonary journals:

IF increased considerably in 2020 compared to 2019 in pulmonary journals. Δ-IF was also significantly higher in 2020 than in 2019 (Tables 1, 2).

## Discussion

This cross-sectional study demonstrated a sudden uptrend in IFs of pediatric journals in 2020 compared to 2019. In contrast, other bibliometrics including IF ranking and Eigenfactor scores, were relatively stable within that time interval. IF and Eigenfactor also did not change meaningfully between 2018 and 2019. However, SNIP scores of pediatric journals progressively increased from 2018 to 2020. We also determined that the uptrend in IF was not limited to the top-ranked pediatric journals; instead, the phenomenon was universally observed irrespective of journals' ranking. A similar trend was also observed in other biomedical fields beyond pediatrics. The scientific community entrusts IF as a reliable tool to estimate journals' significance, and the sudden uptrend might have conveyed biased messages.

Merits of IF and Eigenfactor have been compared in the past. Eigenfactor is an efficient scientific metric since it is also adjusted for citing journals' quality. Journal rankings often change depending on whether IF or Eigenfactor were used (Rizkallah and Sin, 2010), indicating that journals should not be evaluated based on a single metric. Additionally, our study demonstrated an advantage of the Eigenfactor because of its ability to resist year-to-year variation due to increased publication volume. SNIP, designed by Scopus, was primarily intended to adjust for the variation in citation patterns among different scientific fields (Leydesdorff and Opthof, 2010). However, we found that SNIP also became vulnerable like IF to the sudden upsurge in publication numbers.

**Figure 1:**
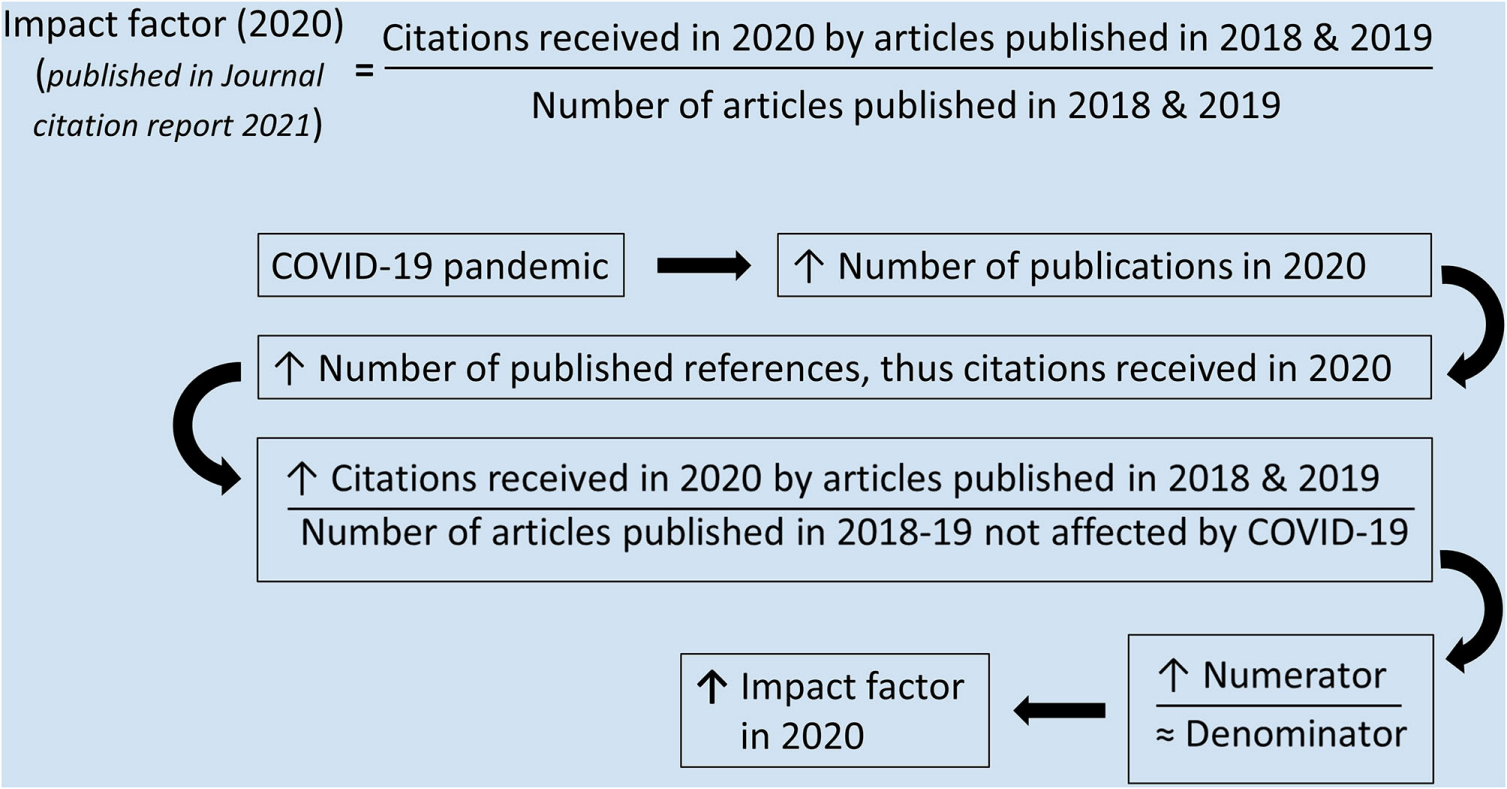
The influence of COVID-19 outbreak on the upsurge of impact factors.

A considerably higher volume of biomedical articles and references were published in 2020 compared to 2019, which perhaps prompted the upsurge of IFs across the various fields of biomedical sciences (Figure 1). The rationale behind increased publication volumes in 2020 was beyond the scope of this study. However, it might be related to markedly escalated research productivity since the COVID-19 outbreak (Nigrovic and Napper, 2021). Both OA and subscription journals experienced a considerable increase in publication volume in 2020. However, the OA journals perhaps were more lenient with acceptance due to lack of space constraints and experienced a higher growth rate than the subscription journals.

Our study has several limitations. This project focused solely on citation counts and not how the journals could influence the IF. For example, some journals no longer publish case reports as those types of articles would count toward the citable papers but are infrequently referenced (Rison et al., 2017). Journals may also prefer to accept reviews (more often cited) or publicize aggressively in social media. Second, we considered all biomedical journals as the source of citations for pediatric journals, which could be an overgeneralization. Moreover, traditional subscription journals often publish few OA articles, as several publishers have been leaning toward a hybrid model in recent years (Pinfield et al., 2017). Thus, OA vs. subscription journal classification could be an oversimplification. Nonetheless, this study has few strengths. To the best of our knowledge, no previous study reported the sudden uptrend in IF since the outbreak of COVID-19. Our study showed that increased IF affected not only all tires of pediatric journals but other specialties too. Henceforth, it is likely to be a general phenomenon. Finally, we analyzed other bibliometrics in addition to IF and found that Eigenfactor scores were more stable than IF.

## Conclusion

A significant upsurge in pediatric journals' IF in 2020 was perhaps related to the sudden increase in overall publication volumes since the COVID-19 outbreak. Due to its vulnerability, IF should be cautiously reported as a benchmark of excellence. Unlike IF, Eigenfactor scores of pediatric journals remained unchanged in 2020, citing an additional advantage of Eigenfactor compared to other bibliometrics. A similar trend in IF was observed among all quartiles of pediatric journals, irrespective of quality and prestige. A similar uptrend in IF was also observed in journals of another specialty such as pulmonology. Both OA and subscription journals experienced a considerable increase in publication volume in 2020. However, the OA journals experienced even higher growth than the subscription journals.

## Data Availability Statement

The data will be made available upon request.

## Author Contributions

PM conceptualized, designed the study, supervised data collection, analyses, drafted the initial manuscript, reviewed, and revised the manuscript. LM, LS, and SG conceptualized, designed the data collection instruments, collected data, conducted initial analyses, reviewed, and revised the manuscript. ED conceptualized the study, supervised various aspects of accessing the data, and critically reviewed the manuscript for important intellectual content. All authors approved the final manuscript as submitted and agree to be accountable for all aspects of the work.

## Conflict of Interest

The authors declare that the research was conducted in the absence of any commercial or financial relationships that could be construed as a potential conflict of interest.

## Publisher's Note

All claims expressed in this article are solely those of the authors and do not necessarily represent those of their affiliated organizations, or those of the publisher, the editors and the reviewers. Any product that may be evaluated in this article, or claim that may be made by its manufacturer, is not guaranteed or endorsed by the publisher.

## Abbreviations

IF: impact factor
OA, open access, JCR: journal citation report
SNIP: source normalized impact per paper
IQR: Interquartile range.
