# Supplementary material for: The Upsurge of Impact Factors in Pediatric Journals Post COVID-19 Outbreak: A Cross-Sectional Study
Source: Front Res Metr Anal. 2022 Mar 29;7:862537. doi: 10.3389/frma.2022.862537 (PMC9002324; doi:10.3389/frma.2022.862537)
Supplement: Supplementary file 1 [file Table_1.pdf]

Supplemental Table 1:

Variables	Statistics	p-value
Impact factor 2020	0.750	<0.001
Eigenfactor Score 2020	0.351	<0.001
SNIP 2020	0.831	<0.001
Impact factor 2019	0.719	<0.001
Eigenfactor Score 2019	0.386	<0.001
SNIP 2019	0.833	<0.001
Impact factor 2018	0.795	<0.001
Eigenfactor Score 2018	0.403	<0.001
SNIP 2018	0.832	<0.001

Shapiro-Wilk tests to analyze the normal distribution of the data on the bibliometrics of the pediatric journals. P-value<0.05 indicates statistically significant result or rejection of the null hypothesis 'that the data are normally distributed'.

Variables	Statistics	p-value
Total publications 2020	0.357	<0.001
Total references 2020	0.203	<0.001
Total publications 2019	0.395	<0.001
Total references 2019	0.256	<0.001
Total publications 2018	0.444	<0.001
Total references 2018	0.308	<0.001

Shapiro-Wilk tests to analyze the normal distribution of the data on the publication and reference counts of the biomedical journals. P-value<0.05 indicates statistically significant result or rejection of the null hypothesis 'that the data are normally distributed'.
